# Evaluation of antibacterial and cytotoxic activity of *Artemisia nilagirica* and *Murraya koenigii* leaf extracts against mycobacteria and macrophages

**DOI:** 10.1186/1472-6882-14-87

**Published:** 2014-03-05

**Authors:** Sumanta Kumar Naik, Soumitra Mohanty, Avinash Padhi, Rashmirekha Pati, Avinash Sonawane

**Affiliations:** 1 School of Biotechnology, KIIT University, Campus-11, Bhubaneswar, Orissa 751024, India

**Keywords:** Tuberculosis, Mycobacteria, Antibacterial activity, Cytotoxicity, Genotoxicity, *Artemisia nilagirica*, *Murraya koenigii*

## Abstract

**Background:**

*Artemisia nilagirica* (Asteraceae) and *Murraya koenigii* (Rutaceae) are widely distributed in eastern region of India. Leaves of *Artemisia nilagirica* plant are used to treat cold and cough by the local tribal population in east India. *Murraya koenigii* is an edible plant previously reported to have an antibacterial activity. Pathogenic strains of mycobacteria are resistant to most of the conventional antibiotics. Therefore, it is imperative to identify novel antimycobacterial molecules to treat mycobacterial infection.

**Methods:**

In this study, ethanol, petroleum ether and water extracts of *Artemisia nilagirica* and *Murraya koenigii* were tested for antibacterial activity against *Mycobacterium smegmatis* and *Mycobacterium bovis* BCG in synergy with first line anti-tuberculosis (TB) drugs, and for cytotoxic activities on mouse macrophage RAW264.7 cells. Antibacterial activity was determined by colony forming unit (CFU) assay. Intracellular survival assay was performed by infecting RAW264.7 cells with *M. smegmatis* before and after treatment with plant extracts. Cytotoxity was checked by MTT [3-(4,5-Dimethylthiazol-2-yl)-2,5-Diphenyltetrazolium Bromide] assay. Genotoxicity was studied by DAPI staining and COMET assay using mouse macrophage RAW264.7 cell line. Cell apoptosis was checked by Annexin-V/FITC dual staining method. Reactive oxygen species and nitric oxide production was checked by DCFH staining and Griess reagent, respectively.

**Results:**

Ethanol extracts of *A. nilagirica* (IC_50_ 300 μg/ml) and *M. koenigii* (IC_50_ 400 μg/ml) were found to be more effective against *Mycobacterium smegmatis* as compared to petroleum ether and water extracts. *M. koenigii* extract showed maximum activity against *M. bovis* BCG in combination with a first line anti-TB drug rifampicin. *M. koenigii* leaf extract also exerted more cytototoxic (IC_50_ 20 μg/ml), genotoxic and apoptosis in mouse macrophage RAW 264.7 cell line. Treatment of mouse macrophages with *A. nilagirica* extract increased intracellular killing of *M. smegmatis* by inducing production of reactive oxygen species and nitric oxide*.*

**Conclusions:**

Ethanol extracts of *A. nilagirica and M. koenigii* were found to be more effective against mycobacteria. As compared to *A. nilagirica*, *M. koenigii* ethanol extract exhibited significant synergistic antibacterial activity against *M. smegmatis* and *M. bovis* BCG in combination with anti-tuberculosis drug rifampicin, and also showed increased cytotoxicity, DNA damage and apoptosis in mouse macrophages.

## Background

The expanding bacterial resistance to antibiotics has become a growing concern worldwide. *Mycobacterium tuberculosis* (Mtb), the causative agent of tuberculosis (TB), infection represents a major health problem globally. It is estimated that one third of the world’s population is infected with TB and 1 in 10 of them is prone to develop an active TB in their lifetime [1]. Though for several decades TB was successfully treated with a combination of antibiotics, current vaccine and chemotherapeutic measures are limited in their efficacy and are failing to prevent spread of the disease. This problem is further worsened due to the emergence of multi-drug resistant (MDR) and extremely drug-resistant (XDR) Mtb strains that has made most of the front line drugs ineffective, too often impossible, to cure fatal infections [2]. MDR-TB is resistant to the major first line anti-TB drugs isoniazid and rifampicin, while XDR-TB is resistant to any fluoroquinolone, and at least one of three injectable second-line drugs (capreomycin, kanamycin, and amikacin), in addition to isoniazid and rifampicin. It is thus important to identify and develop new molecules that can overcome the limitations of the present drugs and perturb development of antibiotic resistance by mycobacteria.

Increasing bacterial resistance is prompting resurgence in research of the antimicrobial role of herbs against resistant strains. A vast number of medicinal plants have been recognized as valuable resources of natural antimicrobial compounds [3,4]. Medicinal plant extracts offer considerable potential for the development of new agents effective against infections that are currently difficult to treat [5,6]. Previous studies have shown that several substances such as peptides, unsaturated long chain aldehydes, essential oils and alkaloid constituents of plant extracts have potential therapeutic properties [7]. Therefore, assessment of such plants remains an interesting and useful task to find new promising agents against bacterial infections. In case of TB, the first drug streptomycin was developed in 1947 and later on isoniazid, pyrazinamide and ethambutol were introduced, and then in 1960’s rifampicin, which till today is considered as the cornerstone of first-line TB drugs. Since then the development of anti-TB drugs has faltered. In the last few decades, though a large number of drugs have been discovered, many of them failed to kill drug resistant bacteria effectively. Moreover, mycobacterium has a lipid rich cell wall, which prevents binding and diffusion of most of the drugs [8]. Therefore, there is a need to identify molecules that will act directly on the cell wall of mycobacteria to achieve effective killing.

*Artemisia nilagirica,* which belongs to family Asteraceae, and *Murraya koenigii* belonging to family Rutaceae are well distributed in eastern region of India. Previously the leaf extract of *A. nilagirica* has been reported with an antibacterial activity [9]. The leaves of this plant are also used against cold and cough by the local tribal population in east India. *M. koenigii* is an edible plant. Compounds like mahanine, mahanimbicine and mahanimbine from *M. koenigii* plant are reported to have antibacterial activity against *Staphylococcus aureus, Pseudomonas aeruginosa, Klebsiella pneumoniae, Escherichia coli and Streptococcus pneumoniae*[10,11].

The main objective of this work was to identify plants with antimycobacterial activity. Here we have screened several leaf extracts prepared from *Eupatorium triplinerve, Nyctanthes arbortristis, Azadirachta indica, Barringtonia acutangula, Achyranthes aspera, Moringa oleifera, Artemisia nilagrica, Murraya koenigii, Lantana camara, Mentha spicata* for their antimycobacterial activity*.* These plants were selected on the basis of their reported edible or medicinal properties by local tribal population of eastern India. Among them, *A. nilagirica* (IC_50_ 300 μg/ml) and *M. koenigii* (IC_50_ 400 μg/ml) ethanol leaf extracts were found to be more effective against *M. smegmatis* and *M. bovis* BCG. Ethanol extract of *M. koenigii* showed increased antibacterial activity against *M. bovis* BCG in synergy with an anti-TB drug rifampicin. *Ex vivo* studies showed that *A. nilagirica* extract is able to kill intracellular *M. smegmatis* in macrophages by inducing the production of reactive oxygen species (ROS) and nitric oxide (NO). Toxicity studies showed that *M. koenigii* ethanol extract exhibit more cytotoxic and genotoxic activities and also induced more apoptosis in mouse macrophage RAW264.7 cell line, which indicates presence of both antimycobacterial and cytotoxic compounds in the extract.

## Methods

### Preparation of plant extract

All the plants were identified by Dr. Udhab Behera from Regional Plant Resource Centre, Odisha and these plants have been deposited in the herbarium at Regional Plant Resource Centre, Keonjhar, Odisha, India. Leaves of *A. nilagirica* (KCCP/2013/AN/102) and *M. koenigii* (KCCP/2013/MK/101) were collected from forests of North Odisha region, India. Leaves were first washed thoroughly to remove impurities, shade dried and then ground to fine powder. To prepare extract, leaf powder (10 gm dry weight) was extracted with 50 ml of absolute ethanol and kept on shaker for 48 hours. The extract was then centrifuged at 2300 g (3-30 K, Sigma) for 20 minutes and supernatant was collected. Solvent was removed with the help of rotary evaporator and stored at −20°C. Extract was dissolved in 1% DMSO (wt/vol) for further use [12].

### Cell lines and bacterial culture conditions

*Mycobacterium smegmatis* mc^2^155 and *M. bovis* BCG Pasteur (ATCC35734) strains were grown in Middlebrook’s 7H9 broth medium (Difco) supplemented with 10% OADC (Oleic acid–albumin dextrose–catalase) and 0.05% tween 80 (Merck) at 37°C and 120 rpm. The mouse macrophage cell line RAW264.7 was cultured in DMEM supplemented with 10% fetal calf serum (FCS), 1% penicillin-streptomycin solution, 1% L-glutamine and HEPES.

### In vitro killing assay

To determine the antibacterial activity of plant extracts, the overnight grown cultures were centrifuged at 2300 g for 5 minutes, washed with 1X PBS and the pellet was suspended in 7H9 broth medium. Finally the optical density (O.D.) of the sample was adjusted to 0.1 at 600 nm that corresponds to 1 × 10^7^ cfu/ml [13]. Various concentrations of plant extracts were incubated with 4-5 × 10^5^ bacteria in 7H9 media. Medium with bacteria and 1% DMSO only were used as controls. Bacteria were harvested at 24 h time point and the number of colony forming units (CFUs) was assayed by plating serially diluted samples on 7H9 medium. The colonies were counted after 72 h and 3 weeks for *M. smegmatis* and *M . bovis* BCG, respectively.

To check the synergistic activity of plant extract and anti-TB drugs, IC50 values of rifampicin (0.7 μg/ml) and isoniazid (7.5 μg/ml) were determined by CFU assay and their synergistic antimycobacterial activity was checked with different concentrations (200 and 400 μg/ml) of plant extracts.

### Isobologram analysis

Isobologram analysis was performed to check the combinational effect of *M. koenigii* and rifampicin. IC50 values obtained from *M.koeingii* (400 μg/ml) and rifampicin (0.7 μg/ml) were analyzed by joining the data points with a line. The IC50 value from the combinatorial treatment was also plotted on the same plot. Isobologram analysis considered the data additive if the data point falls on the line, synergistic interaction appears below the line, and an antagonistic interaction appears above the line [14].

### Cytotoxic effect of plant extracts on mouse macrophage RAW264.7

To determine the cytotoxic activity of plant extracts on macrophages, RAW264.7 in DMEM were grown in a 96 well plate (10,000 cells per well) at 37°C, 5% CO_2_ for 24 hours followed by treatment with different concentrations of plant extracts for another 24 hours. Cells were washed with 1X PBS before addition of MTT. To determine the cell viability, MTT at a concentration of 0.1 μg/ml was added to the wells and incubated for 4 hours at 37°C and 5% CO_2_ in dark condition. In metabolically active cells, MTT reduced to purple color insoluble formazan crystal. Formazan crystals were dissolved in dissolving buffer (11gm SDS in 50 ml of 0.02 M HCl and 50 ml isopropanol). The absorbance was read at 570 nm in ELISA reader (Biotek, Germany), compared with the untreated cells and percentage of viable cells was calculated as described previously [15].

### Apoptosis assay

The percentage of apoptotic cells was determined by using an Annexin V-FITC apoptosis detection kit (Sigma) as described previously [16]. Briefly, 2 × 10^5^ RAW264.7 cells per well were grown overnight in a six-well culture plate, treated with various concentrations of plant extracts and the cells were incubated for another 12 h. The cells were treated with trypsin, washed three times with Dulbecco’s phosphate buffer saline (0.1 M, pH 7.4) and 500 μl of 1X binding buffer was added, followed by 5 μl of annexin V-FITC and 10 μl of propidium iodide, and then incubated for 10 min at room temperature in the dark. Untreated cells were taken as a negative control. Flow cytometry was performed by analyzing 10,000 gated cells using a FACSCalibur flow cytometer and CellQuest software (Becton Dickinson, USA).

### Comet assay

The effect of plant extracts on DNA damage was determined by alkaline single cell electrophoresis (Comet) assay. In 6-well cell culture plate, 5 × 10^5^ RAW264.7 macrophages per well were treated with *M. koenigii* (100 μg/ml) and *A. nilagirica* (300 μg/ml) extracts for 12 h [17]. These concentrations were found to be effective against *M. smegmatis* under *in vitro* killing assay. Untreated cells were used as control. Cells were treated with trypsin and a cell suspension (5000 cells/μl) was prepared in 1X phosphate-buffered saline (PBS). Then, 10 μl of cell suspension was mixed with 60 μl of 0.5% low-melting point agarose. A thin smear of cell suspension was prepared in glass cavity slides (Blue Label Scientifics, Mumbai, India). The agarose was allowed to solidify in the dark at 4°C for 45 min, and then the slides were submerged in lysis solution (10 mM Tris, 100 mM EDTA, 2.5 M NaCl, 1% Triton-X-100, 10% dimethyl sulfoxide) for 30 min in dark at 4°C. After lysis, the slides were washed with distilled H_2_O, transferred to an electrophoresis unit containing freshly prepared electrophoresis buffer (500 mM EDTA, 200 mM NaOH, pH13.0), left for unwinding of DNA for 45 min, and the cells were electrophoresed for 15 min at 15 V. The cells were washed twice with dH_2_O, fixed with 70% chilled ethanol, and stained with 0.5 μg of propidium iodide/ml for 15 min in dark. The slides were dried and observed using a fluorescence microscope (Nikon, Japan).

### Effect on intracellular killing of mycobacteria

To examine whether treatment with plant extracts increase killing efficiency of macrophages, 5 × 10^5^ RAW264.7 cells were treated with *A. nilagirica* extract 2 h before and after *M. smegmatis* infection at an multiplicity of infection of 10 termed as “pretreated” and post-treated”, respectively as described previously [13]. Then the cells were washed with 1X PBS and extracellular bacteria were killed by addition of 20 μg/ml of gentamicin for 1 h. Macrophages infected with bacteria alone were used as a control. After 6 h incubation period, cells were washed and lysed with 0.5% triton X-100. The intracellular survival was determined by plating serially diluted cultures on 7H10 medium and the colonies were enumerated after 3 days.

### Determination of ROS production

Generation of ROS in RAW264.7 was determined by using 2′, 7′-dichlorofluorescin diacetate (DCFH-DA), a lipid-permeable non-fluorescent compound that when oxidized by intracellular ROS in the presence of cellular esterase, forms the fluorescent compound 2′, 7′-dichlorofluorescein (DCF). RAW264.7 cells (2 × 10^5^) were seeded on 24-well tissue culture plate. After 24 h, the cells were treated with different concentrations of *A. nilagirica*. Untreateded cells were used as control. After 24 h of treatment, media was removed and DMEM containing 10 μM DCFH-DA was added to the cells and incubated at 37°C and 5% CO_2_ for 4 h. Then the DCFH-DA containing medium was removed and the cells were rinsed with 1X PBS and the fluorescence intensity of DCF was measured using 10,000 gated cells by flowcytometer BD FACS Canto-II and FACS Diva software as described previously [18].

### Determination of NO production

NO production was determined by using Griess reagent. RAW264.7 cells were treated with different concentrations (100–400 μg/ml) of plant extracts for 24 h. Supernatant was collected and 100 μl of each of them was added in triplicate in a 96-well plate. NaNO_2_ solution (0–200 μM) was used as a standard. 100 μl/well of the Griess reagent (6 mg/ml) was added to the samples and the reaction products were estimated colorimetrically at 550 nm [19].

### Statistical analysis

Statistically significant differences between groups were determined using Mann Whitney test. Significance was referred as ** for P ≤ 0.0005 and * for P ≤ 0.01.

## Results

### Antibacterial activity of plant extracts

**Figure 1:**
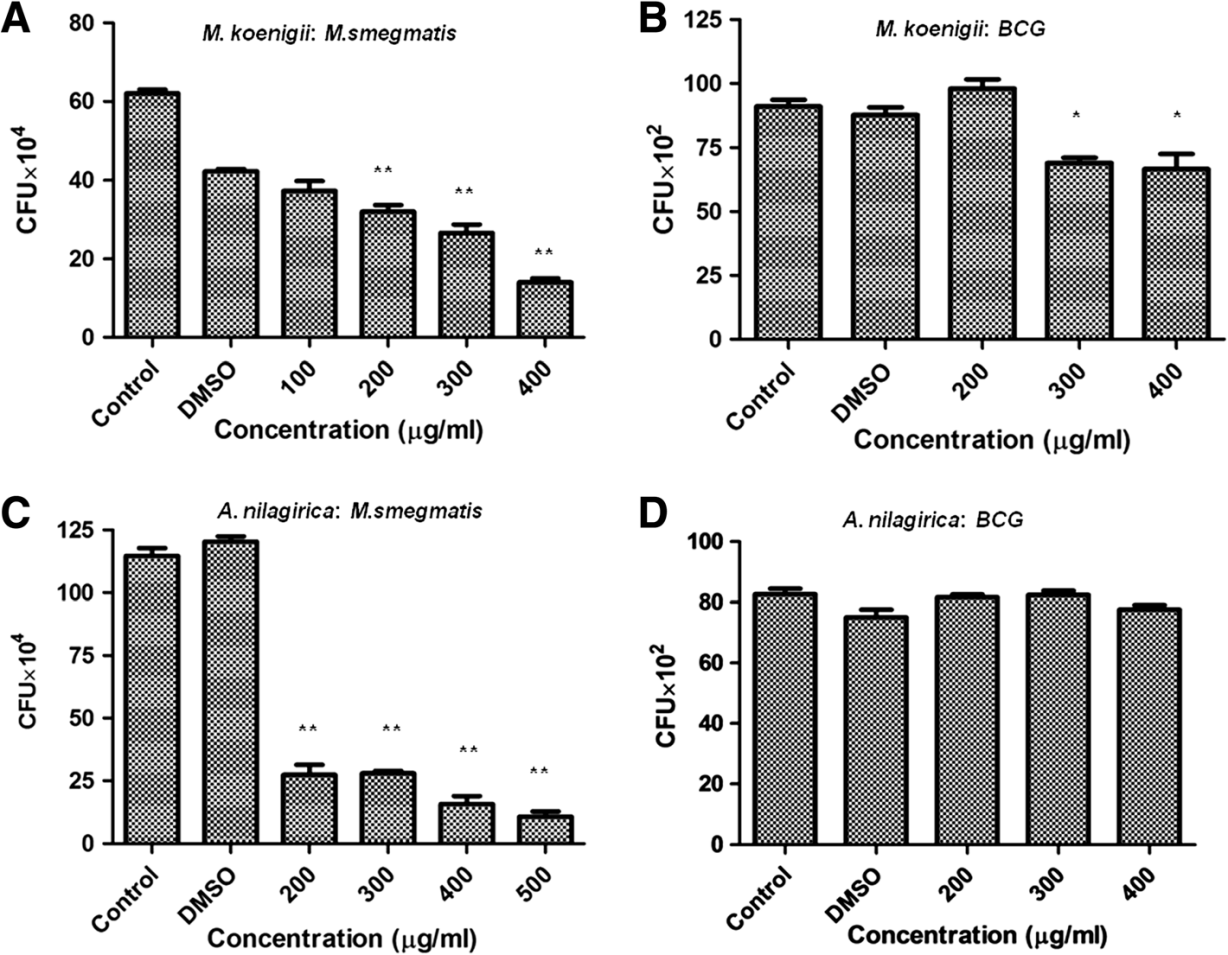
**Antibacterial activity of *M. koenigii *and *A. nilagirica *ethanol leaf extracts against *M. smegmatis *and *M. bovis *BCG.** 4-5 × 10^5^ *M. smegmatis***(A,C)** and *M. bovis* BCG **(B, D)** were incubated with different concentrations of *M. koenigii* and *A. nilagirica* ethanol leaf extracts for 24 h in 7H9 medium and the number of colony forming units were counted after 72 h and 3 weeks for *M. smegmatis* and *M.bovis* BCG, respectively by plating serially diluted samples on 7H9 medium. Media containing bacteria alone was used as control. Experiments were performed in triplicates, means ± the standard deviation (SD) are shown. **,P ≤ 0.0005; *,P ≤ 0.01.

Several plant leaf extracts, *Eupatorium triplinerve, Nyctanthes arbortristis, Azadirachta indica, Barringtonia acutangula, Achyranthes aspera, Moringa oleifera, Artemisia nilagrica, Murraya koenigii, Lantana camara, Mentha spicata* were screened for anti-mycobacterial activity. These plants were identified by Dr. Udhab Behera, Regional Plant Resource Centre, Odisha, India. These plants have been deposited in the herbarium at Regional Plant Resource Centre, Keonjhar, Odisha, India. Extracts were prepared using petroleum ether, ethanol and water solvents. Among above mentioned plants, only ethanol extracts of *A. nilagirica* and *M. koenigii* showed antibacterial activity against *M. smegmatis,* as determined by CFU assay. However, extracts prepared using petroleum ether and water did not show any anti-mycobacterial activity (data not shown). As shown in Figure 1, ethanol leaf extracts of *M. koenigii* and *A. nilagirica* showed significant antibacterial activity at 400 μg/ml (P ≤ 0.0005; Figure 1A) and 200 μg/ml (P ≤ 0.0005; Figure 1C) concentrations against *M. smegmatis*, respectively. However, *M. bovis* BCG was found to be resistant to both the plant extracts under similar conditions (Figure 1B and 1D). As the ethanol extract showed more antibacterial activity following experiments were performed with *M. koenigii* and *A. nilagirica* ethanol extracts only*.*

### Synergistic antibacterial activity of plant extracts and anti-TB drugs

**Figure 2:**
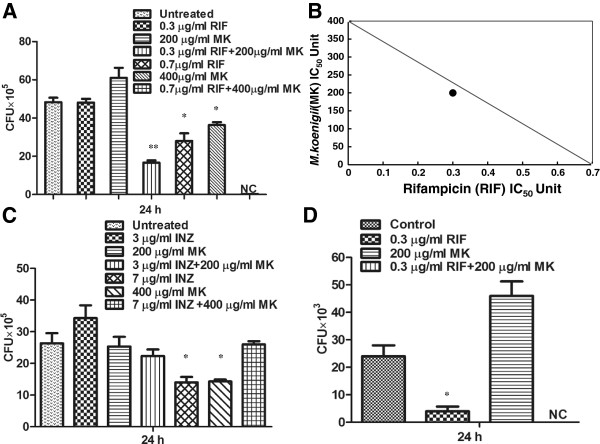
**Synergistic antibacterial activity of *M. koenigii *(MK) ethanol leaf extract and first-line anti-TB drugs. (A)***M. smegmatis* was incubated with MK extract (200 and 400 μg/ml) alone or in combination with rifampicin (RIF) (0.3 and 0.7 μg/ml) for 24 h. The number of CFUs were counted after 72 h of incubation. Data showed decreased bacterial count in presence of both MK and RIF. **(B)** Isobologram analysis of MK extract and RIF. The data point falls below the line indicating syngergistic activity. **(C)***M. smegmatis* was incubated with MK extract and isoniazid (INZ) for 24 hours and numbers of CFUs were assayed. **(D)***M. bovis* BCG was incubated with MK extract (200 μg/ml) and rifampicin (RIF) (0.3 μg/ml) for 24 hours and numbers of CFUs were assayed after 3 weeks. Media containing bacteria alone was used as control. NC means no colonies. Experiments were performed in triplicates, means ± the SD are shown. **,P ≤ 0.0005; *,P ≤ 0.01.

**Figure 3:**
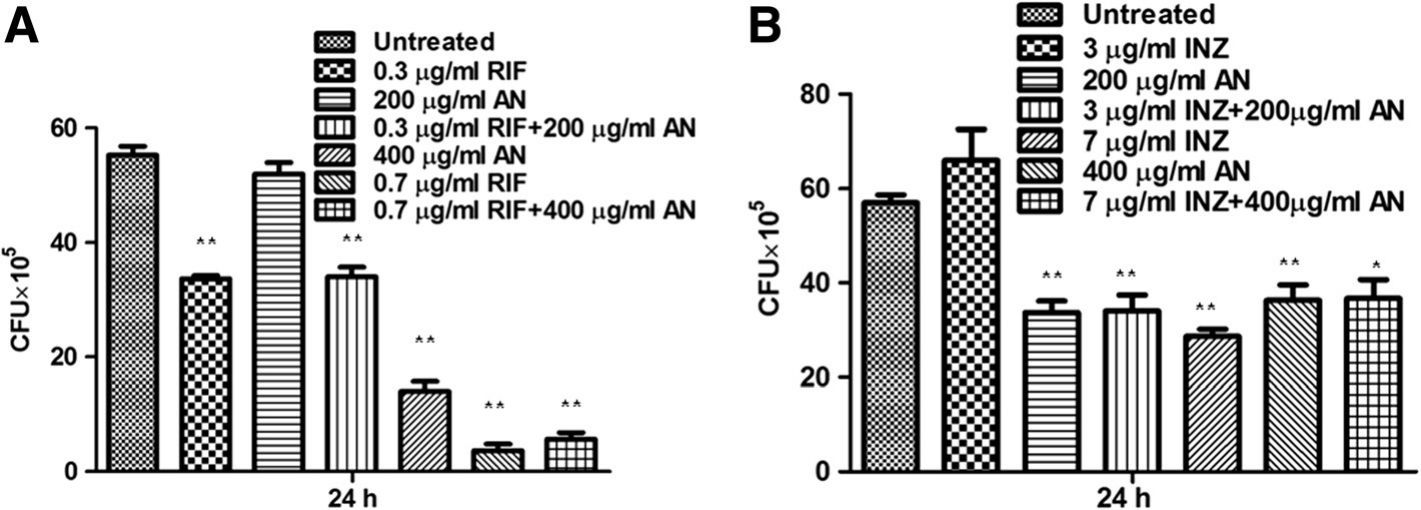
**Synergistic anti-bacterial activity of *A. nilagirica *(AN) ethanol leaf extract and first-line anti-TB drugs.***M. smegmatis* was incubated with **(A)** AN extract and rifampicin (RIF) **(B) **AN extract and isoniazid (INZ) for 24 hours and number of CFUs were assayed. Data showing no synergism of *A. nilagirica* with either of the drugs. Media containing bacteria alone was used as control. Experiments were performed in triplicates, means ± the SD are shown. **,P ≤ 0.0005; *,P ≤ 0.01.

As *M. bovis* BCG showed resistance to both the plant extracts, we checked whether addition of anti-TB drugs rifampicin and isonizid to plant extracts can enhance the bacterial killing. For this, first IC50 values of rifampicin and isoniazid against *M. smegmatis* were determined. The values were determined as 0.7 μg/ml and 7.5 μg/ml for rifampicin and isoniazid, respectively (data not shown). *M. smegmatis* was incubated with different concentrations of plant extracts containing rifampicin (0.7 μg/ml) and isoniazid (7.5 μg/ml). *M. koenigii* (MK) extract showed synergistic activity with rifampicin (P ≤ 0.0005, P ≤ 0.01; Figure 2A) such that no viable bacteria were observed in presence of rifampicin and 400 μg/ml of *M. koenigii* extract, whereas in presence of MK and rifampicin alone significantly higher bacterial count was observed. Isobologram analysis showed that the data point for the combination of *M. koenigii* (MK) and rifampicin(RIF) falls below the line, indicating synergistic activity (Figure 2B). No synergistic activity was observed with isoniazid (Figure 2C). As MK extract showed synergistic activity with rifampicin, we checked their activity against *M. bovis* BCG. No viable colonies were observed in presence of a combination of MK and rifampicin, whereas significantly higher bacterial colonies were observed in presence of MK extract and rifampicin alone (P ≤ 0.01; Figure 2D). In contrast, *A. nilagirica* (AN) extract did not show any synergistic activity with both rifampicin and isoniazid (P ≤ 0.0005, P ≤ 0.01; Figure 3A and 3B).

### Cytotoxic and genotoxic effect of plant extracts on mouse macrophages

**Figure 4:**
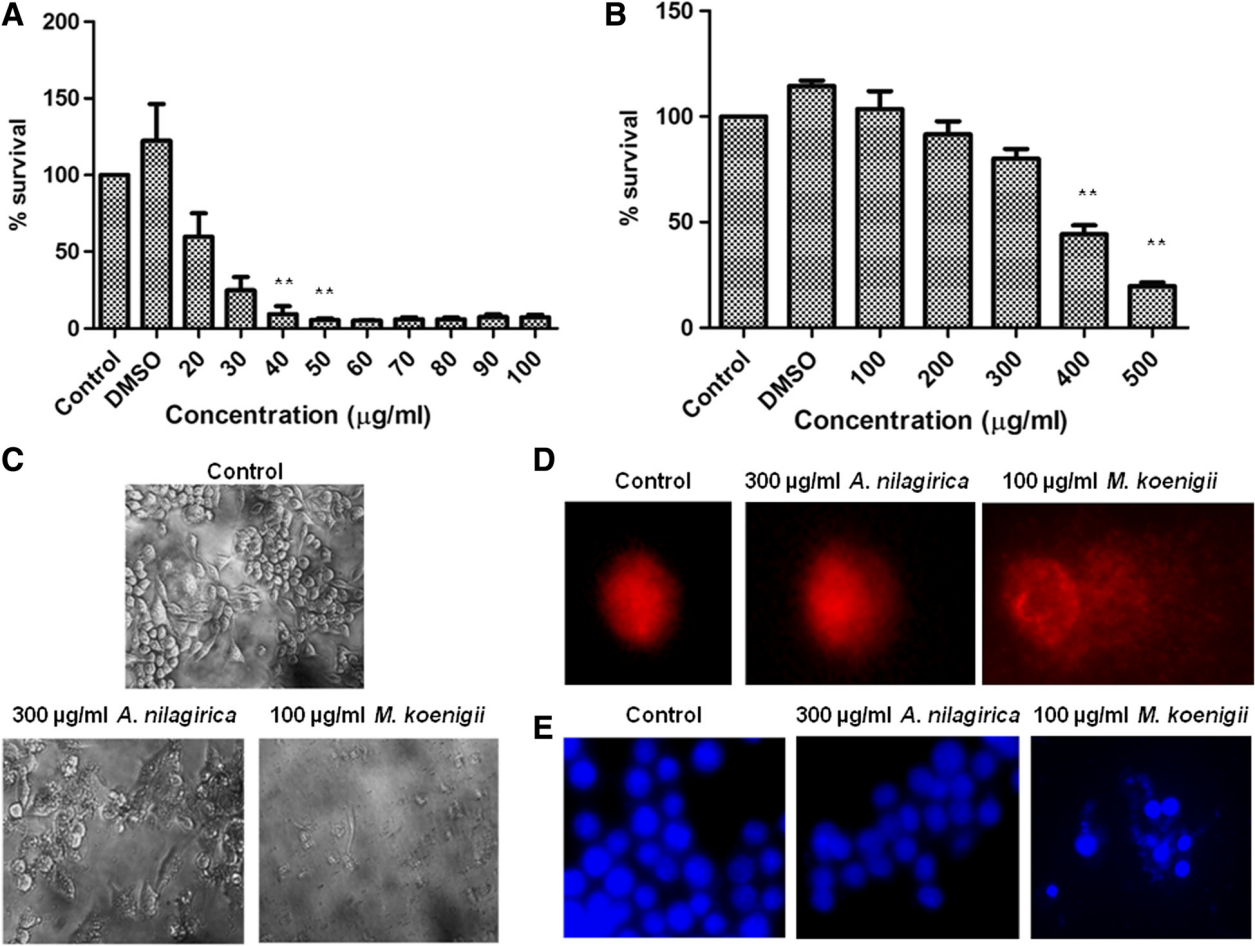
**Cytotoxic effect of *M. koenigii *and *A. nilagirica *extracts on mouse macrophage.** RAW264.7 (1 × 10^4^ cells/ml) in DMEM were grown in a 96 well plate at 37°C, 5% CO_2_ for 24 hours, followed by treatment with different concentrations of *M. koenigii***(A)** and *A. nilagirica***(B)** extracts for another 24 hours. Untreated cells were used as control. Cell viability was determined by MTT assay, *M. koenigii* extract showed more cytotoxic effect as compared to *A. nilagirica* ethanol extract. **(C)** Monolayer images of RAW264.7 cells after treatment with *M. koenigii* and *A. nilagirica* extacts. **(D,E)** Effect of *M. koenigii* and *A. nilagirica* extracts on DNA damage of mouse macrophage. RAW264.7 cells were treated with *M. koenigii* (100 μg/ml) and *A. nilagirica* (300 μg/ml) for 12 h. The effect on DNA damage **(D)** and nuclear fragmentation **(E)** was studied by comet assay and DAPI staining, respectively. Treatment with *M. koenigii* ethanol extract caused DNA damage and nucleus fragmentation in macrophages. Untreated cells were used as a control. Experiments were performed in triplicates; means ± the SD are shown. **,P ≤ 0.0005.

The cytotoxic effect of plant extracts on macrophages was determined by MTT assay, which relies on the fact that metabolically active cells reduce MTT to purple formazan. As shown in Figure 4, *M. koenigii* extract exhibited more cytotoxicity such that at 20 μg/ml concentration approximately 50% reduction in cell viability was observed (P ≤ 0.0005; Figure 4A). In case of *A. nilagirica,* 50% reduction in cell viability was observed at 500 μg/ml (Figure 4B). These data indicated that *M. koenigii* is more cytotoxic as compared to *A. nilagirica*.

We also examined the RAW264.7 morphology in a monolayer culture after treatment with different concentrations of plant extracts. Microscopic observations showed that treatment with 100 μg/ml of *M. koenigii* extract disintegrated the cell morohology. In case of *A. nilagirica* no distinct morphological changes were observed at same concentration, however cells appeared slightly rounded at 300 μg/ml concentration (Figure 4C).

For genotoxic studies, RAW264.7 cells were treated with *M. koenigii* (100 μg/ml) and *A. nilagirica* (300 μg/ml) extracts. Nuclear integrity and DNA damage was studied by DAPI and comet assays. Nuclear fragmentation and comet like tail, which implies DNA damage, was observed in *M. koenigii* treated macrophages, whereas no such DNA damage was observed in *A. nilagirica* treated cells (Figure 4D and 4E).

### Cell apoptosis

**Figure 5:**
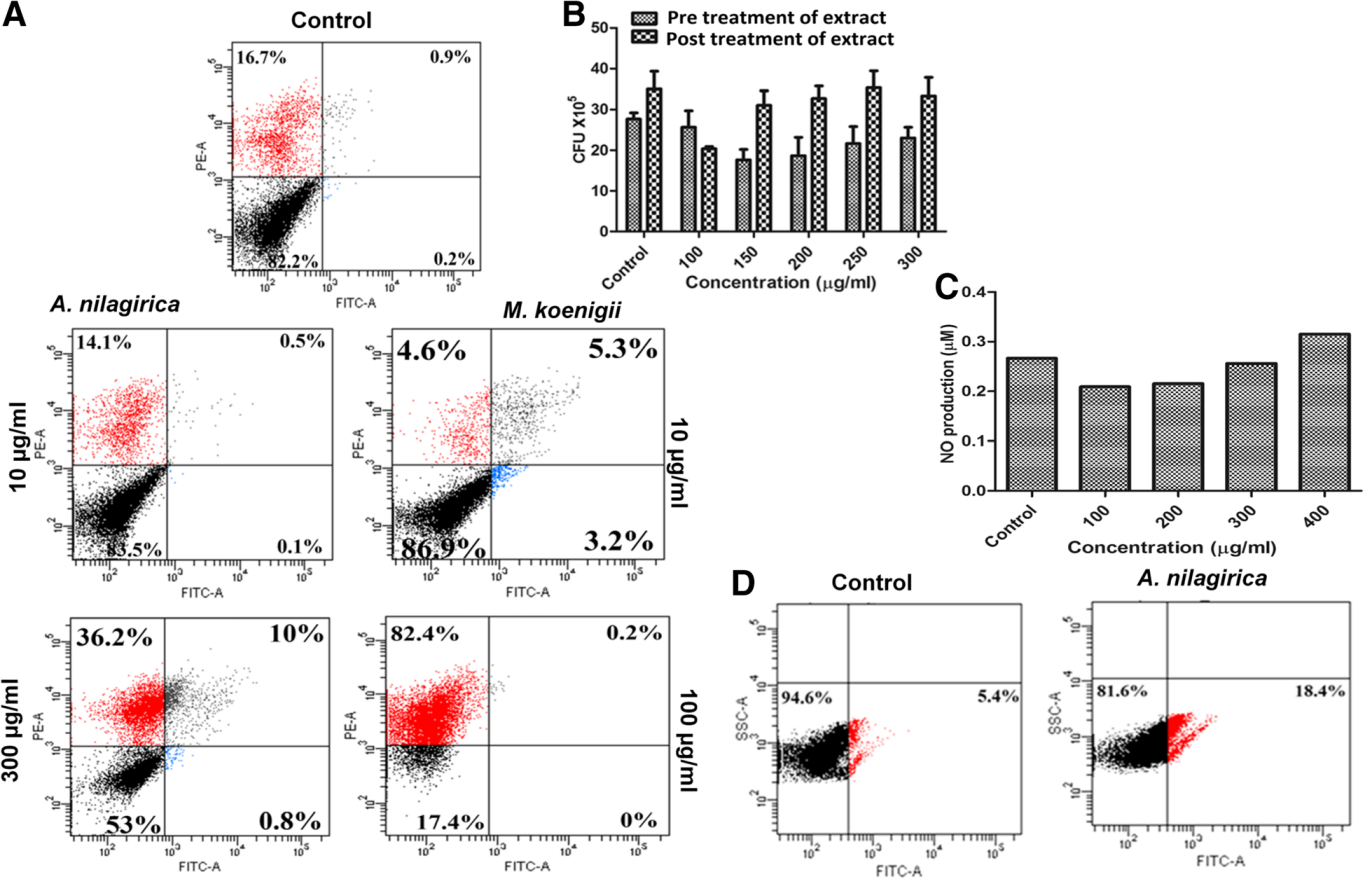
**Apoptosis assay and superoxide production in RAW 264.7 cells. (A)** Apoptosis assay using annexin-V FITC/PI dual staining method. RAW264.7 were treated with *M. koenigii* and *A. nilagirica* extacts for 24 h. The percentage of apoptosis was determined by analyzing 10,000 gated cells using flow cytometry. **(B)** Intracellular survival of *M. smegmatis* in *A. nilagirica* extract treated macrophages. RAW264.7 cells were treated with different concentrations of *A. nilagirica* extacts 2 h before (Pretreatment) and after (post-treatment) *M. smegmatis* infection. The cells were lysed and intracellular survival was determined 6 h post infection by CFU assay. Data showed decrease in intracellular bacterial survival in pretreated macrophages. **(C)** NO production in RAW264.7 treated with different concentrarions of *A. nilagirica* leaf extract for 24 h **(D)** ROS production was determined by DCFH-DA staining using flow cytometry indicating increased ROS production. Experiments were performed in triplicates.

To examine the nature of cell death, annexin-V-FITC/PI dual staining assay was performed according to the manufacturer’s instruction. The dual staining method differentiates early apoptotic (annexin-V positive, PI negative), late apoptotic (both annexin-V and PI positive), necrotic (only PI positive) and healthy viable cells (both annexin-V and PI negative) based on the staining pattern. RAW264.7 cells were treated with *M. koenigii* (10 and 100 μg/ml) and *A. nilagirica* (10 and 300 μg/ml) extracts for 24 h. The data showed that more than 80% cells were viable after treatment with 10 μg/ml concentration of *M. koenigii*, while at 100 μg/ml concentration more than 80% cells were found to be necrotic (Figure 5A). In comparison, no apoptosis was observed after treatment with 10 μg/ml concentration, and significantly less (10%) apoptosis was induced after treatment with 300 μg/ml of *A. nilagirica* extract.

### Intracellular killing of mycobacteria in extract treated macrophages

As *A. nilagirica* leaf extract was found to be less cytotoxic, we treated RAW264.7 cells with *A. nilagirica* leaf extract to check the intracellular killing of mycobacterium. We hypothesized that the extract may stimulate macrophages, which will lead to intracellular killing of mycobacteria. To study this, cells were infected with *M. smegmatis* two hours before and after infection termed as “pretreated” and post-treated”, respectively. After 6 hour of treatment we found that the intracellular bacterial burden was significantly less in pretreated cells in compared to post treated cells (Figure 5B).

### Treatment with *A. nilagirica* extract induce superoxide radical production in macrophages

*Mycobacterium tuberculosis* persists in macrophages for extended period of time. However, upon activation macrophages kill intracellular mycobacteria by the production of superoxide radicals such as ROS, reactive nitrogen species (RNS) and nitric oxide (NO). It was observed that treatment with *A. nilagirica* leaf extract induced the production of NO (Figure 5C) and ROS (Figure 5D) as compared to control cells. These results indicate that the antibacterial activity of *A. nilagirica* leaf extract may be due to inducing the oxidative stress by increasing the production of ROS and NO.

## Discussion

Treatment of drug resistant mycobacterial strains has become a major problem worldwide. The hydrophobic nature of *Mycobacterium tuberculosis* cell wall prevents binding and diffusion of drugs [20]. Moreover, the cell wall of pathogenic mycobacteria contains many efflux pumps, which expels the drugs rapidly before they reach to the site of action [21]. These two, along with several other factors [22], play a decisive role in the development of resistance in mycobacteria. In this study edible and medicinally important plants, few of them are used for the treatment of several diseases by local tribal populations of India, were screened for their anti-mycobacterial activity. Ethanol extracts prepared from *M. koenigii* and *A. nilagirica* plants showed significant antimycobacterial activity. Previous studies have reported anti-inflammatory, anti-bacterial, anti-tumor activities of *M. koenigi* extracts [23,24]. Koenimbine, mahanimbine, mahanine and mahanimboline present in *M. koenigi* extract showed anti-diarrhea and antibacterial activities [25,26]. *A. nilagirica* has been reported to exhibit insecticidal and antibacterial activities [27]. Around 59 compounds were identified from *A. nilagirica* which showed an inhibitory activity against phytopathogens like *Xanthomonas campestris*, *Pseudomonas syringae*, *Clavibacter michiganense*[28].

*In vitro* killing assay using ethanol extracts of *M. koenigi* and *A. nilagirica* showed a significant level of inhibition against *M. smegmatis*. On the other hand, petroleum ether and water extracts did not show any antibacterial activity indicating that antimycobacterial compounds are mainly present in ethanol extract. The *in vitro* antibacterial assay showed that *A. nilagirica* extract is more active against *M. smegmatis*. Previously, phytochemical analysis of *A. nilagirica* ethanol extract showed the presence of alkaloids, phenolic, quinines and saponins, all of which have been shown to possess antimicrobial activity [28]. Distinct differences in the susceptibility to both plant extracts were observed. *M. smegmatis* was found to be more susceptible, whereas the mycobacterial vaccine strain, *M. bovis* BCG showed resistance to both plant extracts. This may due to structural and compositional differences in the cell wall of two groups, which limits the diffusion of drugs inside the bacterial cell. However, treatment with a combination of rifampicin and *M. koenigii* extract led to increased killing of BCG. Rifampicin kills mycobacteria by binding to the β subunit of the RNA polymerase thus interfere in the RNA synthesis. So treatment with *M. koenigii* extract might have facilitated the transport of rifampicin inside the mycobacterial cell by altering cell membrane permeability and thereby killing of mycobacteria by inhibiting RNA synthesis.

Cytotoxic and genotoxic studies showed that *M. koenigii* is more toxic to the mouse macrophages. It was found that with increasing concentrations, percentage of cell viability decreased. This cytotoxic effect could be due to presence of alkaloids in the *M. koenigii* extract. Previous studies have shown that mahanimbine, mahanine, mahanimbicine alkaloids present in *M. koenigii* extract exhibit cytotoxic activity against MCF-7, P388 and HeLa cell lines [11]. This could also be attributed to higher production of oxygen radicals, disintegration of membrane integrity and cytoskeletal functions as a result of treatment. Microscopic studies also showed that treatment with *M. koenigii* extract disintegrated the cell morphology. Selectivity index (SI) is calculated as: SI = Ratio of toxicity to activity = IC50/MIC. For *A. nilagirica*, SI = 500/200 = 2.5 and for *M. koenigii*, SI = 20/400 = 0.05. More SI value indicates more selectivity for bacteria and less cytotoxic for cell line. Selective index of ethanol extracts of *M. koenigii* is less than *A. nilagirica* ethanol extract, which indicates that ethanol extract of *M. koenigii* is less selective for *M. smegmatis* as compared to *A. nilagirica* and toxic for RAW264.7 cell line.

The comet assay showed significant increase in tail length, which manifests DNA damage, after treatment with *M. koenigii* extract*,* whereas treatment with *A. nilagirica* did not show any effect. It has been observed that exposure to plant extracts lead to an increase in DNA damage and necrosis [29].

No cell apoptosis was observed at the lower dose (10 μg/ml), whereas treatment with higher dose of *M. koenigii* (100 μg/ml) resulted in an increase in the number of apoptotic cells. In contrast, significantly less apoptosis was observed in *A. nilagirica* extract treated cells*.* The increased DNA damage and apoptosis at higher doses could also be due to increased production of reactive oxygen species (ROS). It has been reported that increased ROS production caused genotoxic effect in fibroblast cells [30], which may lead to cell apoptosis.

Since mycobacteria are an intracellular pathogen [31,32], which resides in phagosomal compartment of macrophages, it is important to activate the macrophages or deliver the therapeutic molecules to the target sites that would kill the intracellular mycobacteria. Exogenous addition of *A. nilagirica* extract found to kill intracellular *M. smegmatis,* Macrophages kill mycobacteria by inducing the ROS and NO production. We observed increased ROS and NO production in macrophages after treatment with *A. nilagirica* extract. Hence the observed killing effect could be due to formation of superoxide radicals and activation of macrophages. Treatment with plant extract also induces the production of pro-inflammatory cytokines [33], which activate the cells resulting in an increase in killing efficiency of macrophages.

## Conclusions

Here we have shown that ethanol extracts of *M. koenigii* and A*. nilagirica* exhibit high inhibitory potency against *M. smegmatis* and that *M. koenigii* extract in synergy with rifampicin killed *M. bovis* BCG efficiently. *M. koenigii* extract was found to be more cytotoxic and genotoxic to mouse macrophages, whereas *A. nilagirica* extract killed intracellular mycobacteria without exhibiting any toxic effect on macrophages. The intracellular killing could be due to increased ROS and NO production. This will help in development of natural antimicrobials to decrease the side effects of synthetic drugs. Investigation on fractionation and characterization of these active compounds from both plant extracts is under way.

## Abbreviations

MK: *Murraya koenigii*; AN: *Artemisia nilagirica.*

## Competing interests

The authors have no conflict of interests.

## Authors’ contributions

SKN carried out the leaf extraction, antibacterial assay, analyzed the data and drafted the manuscript. AP and RP carried out the cell culture and microscopy experiments. SM performed apoptosis assay and participated in the design of the study and performed the statistical analysis. AS conceived of the study, and participated in its design and coordination and wrote the manuscript. All authors read and approved the final manuscript.

## Pre-publication history

The pre-publication history for this paper can be accessed here:

http://www.biomedcentral.com/1472-6882/14/87/prepub
